# Neuropeptide Regulation of Aquaporin Intracellular Trafficking in Marine Teleost Rectal Enterocytes

**DOI:** 10.3390/ijms27219427

**Published:** 2026-10-22

**Authors:** François Chauvigné, Daniel Toala-Franco, Àngel Rey-Alfonso, Marc Catalán-García, Roderick Nigel Finn, Joan Cerdà

**Affiliations:** 1 Institute of Marine Sciences, Spanish National Research Council (CSIC), 08003 Barcelona, Spain; chauvigne@icm.csic.es (F.C.); angelrey@icm.csic.es (À.R.-A.); nigel.finn@bio.uib.no (R.N.F.); 2 Institute of Biotechnology and Biomedicine (IBB), Universitat Autònoma de Barcelona, Bellaterra, 08193 Cerdanyola del Vallès, Spain; danti82.dtf@gmail.com (D.T.-F.); marccatalangarcia@gmail.com (M.C.-G.); 3 Department of Biological Sciences, University of Bergen, 5006 Bergen, Norway

**Keywords:** vasopressin, oxytocin, phosphorylation, osmoregulation

## Abstract

In marine teleosts, the neuroendocrine regulation of aquaporin intracellular trafficking in osmoregulatory organs such as the intestinal rectum remains underexplored. By using paralog-specific antibodies, here we determined the subcellular localization of seven aquaporin and aquaglyceroporin paralogs in the rectal enterocytes of the marine teleost gilthead seabream (*Sparus aurata*) and investigated the neuropeptinergic effects of arginine vasopressin (Avp) and oxytocin (Oxt) on channel trafficking. The data reveal that Aqp1aa, -1ab2 and -3a are mainly localized in the cytoplasm and basolateral membranes of the enterocytes, while Aqp1ab1, -10bb and -14 are cytoplasmatic and also present in the apical plasma membrane microvilli. In contrast, Aqp0a localizes in the enterocyte subapical cytoplasm. Treatment of rectal explants with Avp in vitro increases the targeting of Aqp1ab1, -1ab2, -10bb and -14, but not of Aqp0a, -1aa and -3a, to the enterocyte apical microvilli, which involves protein kinase A (PKA)-mediated phosphorylation of the channel subdomains. In addition, we find that the differential interaction of the Aqp1ab-type paralogs with teleost-specific 14-3-3ζ-like binding proteins is associated with the trafficking of the channels to the apical microvilli membranes. Conversely, Oxt treatment does not affect the subcellular distribution of any of the investigated aquaporins, except Aqp14, which is internalized in response to the neuropeptide by a partially independent protein kinase C pathway. These data suggest that Avp-PKA signaling controls plasma membrane trafficking of several seabream rectal aquaporins, possibly to facilitate water and solute fluxes across the enterocyte membrane.

## 1. Introduction

Intestinal water absorption is essential for the survival of marine teleosts in the hyperosmotic oceanic environment [1]. To prevent dehydration due to the passive loss of water and gain of ions, marine teleosts imbibe relatively large amounts of seawater [2]. After drinking, the gastrointestinal tract functions as an osmoregulatory organ to support extracellular and intracellular fluid homeostasis through water absorption. This is achieved through active desalination in the water-impermeable esophagus, and further monovalent ion transport coupled to water absorption in the remaining intestinal segments, particularly in the posterior region and rectum [3,4,5]. In these latter epithelial enterocytes, aquaporin water channels are hypothesized to be key regulators of plasma membrane permeability [4,6,7,8]. However, which piscine channels perform this function and how endocrine pathways regulate their cellular and subcellular localizations are not well known.

Aquaporins are a superfamily of integral channel proteins organized as homotetramers in the cell membrane, in which each monomer forms a pore to facilitate transmembrane bidirectional flow of water (classical aquaporins) or of water and other uncharged small solutes, such as glycerol and urea (aquaglyceroporins), down their concentration gradients [9,10]. Topologically, most aquaporins share a common structure with cytosolic N- and C-termini, six α-helices, and five connecting loops (A, B, C, D, and E), with two highly conserved asparagine-proline-alanine (NPA) motifs in the second and fifth loops. These motifs interact near the center of the molecule and combine with a constriction region typically composed of aromatic, arginine residues (ar/R) in the outer channel vestibule to selectively filter water and/or solute permeability while excluding protons [11,12]. According to phylogenetic, genomic and functional analyses, the genomes of jawed vertebrates can encode up to 17 aquaporin subfamilies (AQP0-16) [13,14,15,16,17]. Furthermore, as a result of independent whole-genome duplication events and lineage-specific tandem duplications, teleost fishes harbor a greater diversity of aquaporin paralogs (up to 19–25 and 37–47, in diploids and tetraploids, respectively), potentially allowing neofunctionalization or subfunctionalization of the additional channels for various physiological adaptations [13,14,15,17]. For example, among the recently discovered tandem duplicates, the Aqp1ab-like paralogs occur alongside *aqp1aa* in the teleost-specific TSA1C locus (gene order: *aqp1aa*-*aqp1ab2*-*aqp1ab1*), and the encoded channels contribute to the water uptake that hydrates pelagic eggs in marine fishes [18,19,20]. Other aquaporin clusters (*aqp3a1*-*aqp3a2*; *aqp3b1*-*aqp3b2*; *aqp8aa*-*aqp8ab*; *aqp8ba*-*aqp8bb*; *aqp10aa*-*aqp10ab*; *aqp10ba*-*aqp10bb*) are also prevalent in the actinopterygian lineage [13,17,18,20].

In mammals, transepithelial water transport in the absorptive epithelial enterocytes seems to be mainly mediated by AQP1, -3, -4, -7, -8, -9, -10 and -11 [21,22,23]. In comparison, the same mechanism in marine/euryhaline teleosts is accomplished through subtype-specific paralogs of AQP1, Aqp1aa (formerly termed Aqp1a) and Aqp1ab1 (formerly termed Aqp1b/Aqp1ab), and AQP8 (Aqp8aa/8ab/8bb), located in the apical and basolateral domains of the enterocyte plasma membrane [24,25,26,27], as well as of the aquaglyceroporins AQP3 and -10, although the latter with a minor role [1,28]. On average, the human intestine can osmotically reabsorb about 10 L of fluid per day [29], while the marine teleost intestine can handle an equivalent volume due to its drinking rate (1–5 mL kg^−1^ h^−1^) [4].

The expression patterns of marine teleost aquaporins in the distal portions of the intestine and rectum, in addition to their presence in the enterocyte apical brush border (microvilli) plasma membranes and basolateral regions, suggest a transcellular pathway to maximize intestinal water absorption during salinity adaptation [1,4,28]. In this mechanism, neurohypophysial hormones arginine vasopressin (Avp, formerly known as vasotocin) and oxytocin (Oxt, formerly known as isotocin) are presumed to play a role in acute and long-term adaptation of teleosts to external salinity changes [8,30]. Evidence for the potential role of Avp and Oxt in aquaporin-mediated intestinal water uptake in marine teleosts arises from observations that their cognate receptors are respectively expressed in anterior and rectal enterocytes, where abundant expression of certain aquaporins has been noted or displays increased levels when fish are exposed to high salinities [24,25,26,27,31,32,33]. In the intestinal and rectal epithelium of the marine teleost gilthead seabream (*Sparus aurata*), the localization of AQP1-type water-selective channels (Aqp1aa and -1ab1) and Aqp14 has been reported [16,27], while the presence of other aquaporin paralogs is unknown. Experiments using the heterologous expression of Aqp1aa, -1ab1, -1ab2 and -14 in *Xenopus laevis* oocytes have revealed the differential regulation of the trafficking of these channels by Avp and Oxt [16,19,32]. However, knowledge of the capacity of Avp and Oxt to stimulate aquaporin-mediated intestinal fluid absorption in marine teleosts in vivo, particularly in the rectum, remains limited.

Neurohormone-mediated trafficking and sorting of aquaporins require post-translational modifications (PTMs), with phosphorylation/dephosphorylation of selected residues being the most common mechanism [21]. In this respect, protein kinase A (PKA), protein kinase C (PKC) and mitogen-activated protein kinase (MAPK) pathways have been shown to activate the phosphorylation of C-terminal and loop D serine or threonine residues in some teleost aquaporins using activators of PKA and PKC, such as forskolin (FSK) and phorbol 12-myristate 13-acetate (PMA), respectively [16,18,19,34,35]. In seabream oocytes, systemic and paracrine Avp signaling regulates protein–protein interactions between the loop D and C-terminal subdomains of Aqp1ab-type channels and teleost-specific 14-3-3ζ-like binding proteins (tyrosine 3-monooxygenase/tryptophan 5-monooxygenase activation proteins) through activation of the PKA pathway [18,19]. As a consequence of this PKA-dependent regulation, the Aqp1ab paralogs are directed to different microvillar domains in the oocyte rather than accumulating in a shared membrane region [18,19,20]. Conversely, Oxt may potentially regulate the recycling of Aqp1aa from the follicular epithelial plasma membrane, allowing the Avp-mediated trafficking of the Aqp1ab-type channels to the thecal, granulosa and oocyte plasma membranes to accelerate water influx for the egg hydration process [18,19,20].

In the gilthead seabream, the intestine shows a functional anterior-posterior specialization, where the rectum is more active at water absorption than the anterior intestine in response to external salinity [36]. In addition, previous studies have shown that Avp receptors are most abundant in the rectum, where they can regulate ion absorption [37]. Therefore, to advance our understanding of the water-absorptive function of the marine teleost intestinal rectum, in this study we investigated the broader diversity of aquaporins expressed in seabream rectal enterocytes and experimentally determined the neuroendocrine regulation of their trafficking in rectal explant cultures.

## 2. Results

### 2.1. Multiple Aquaporin Paralogs Are Differentially Distributed in Seabream Rectal Enterocytes

**Figure 1:**
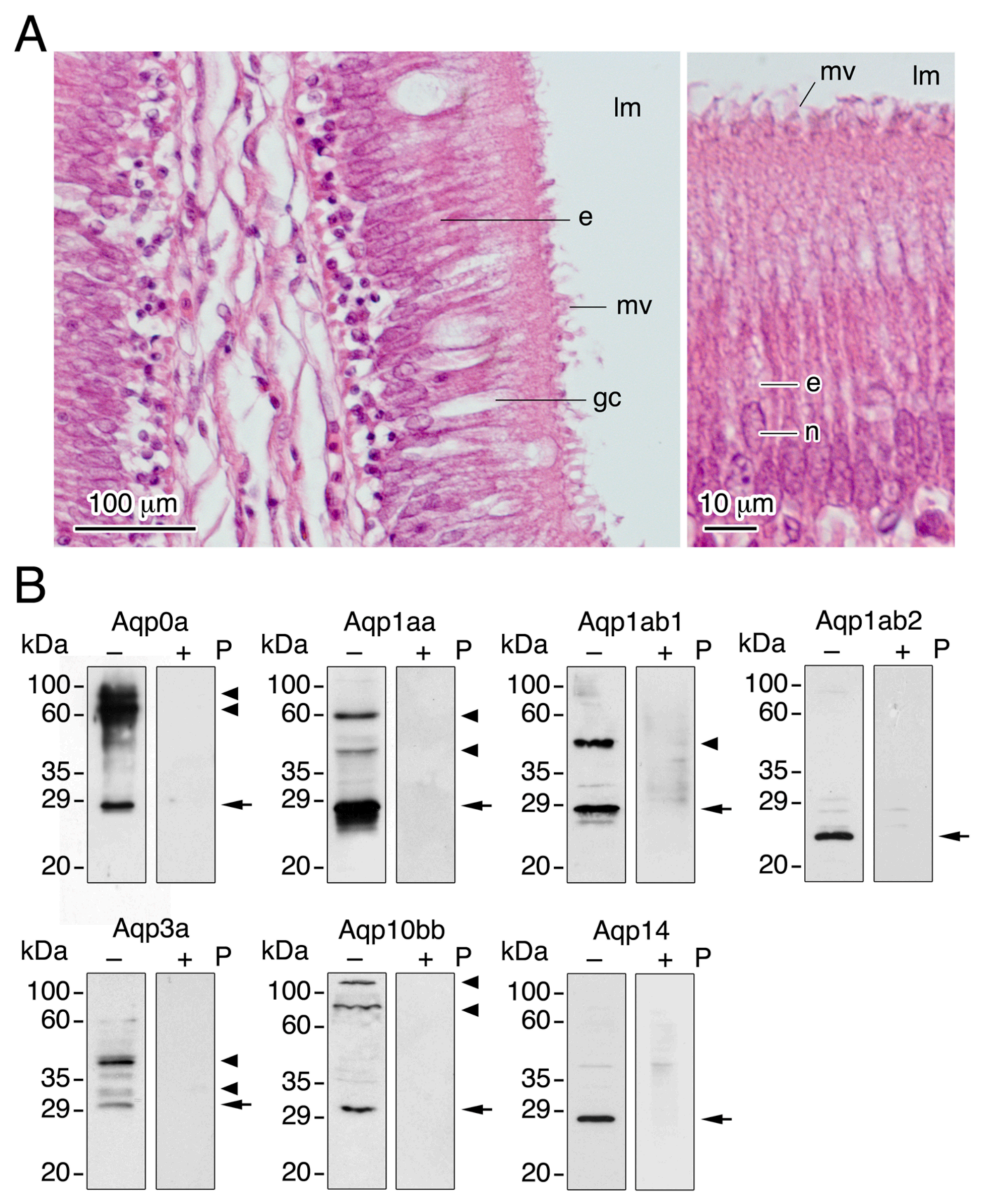
Multiple aquaporin paralogs are expressed in seabream rectal enterocytes. (**A**) Histological section of the rectum stained with hematoxylin and eosin, showing the structure of the enterocytes (**right panel**). lm, lumen; e, enterocyte; n, nucleus; mv, microvilli; gc, goblet cells. (**B**) Immunoblot analysis of aquaporins in total membrane protein extracts from enterocytes using paralog-specific antibodies. Duplicated blots were run in parallel, where incubation was performed using primary antibodies that had been preadsorbed (P) by the antigenic peptides to test for specificity. Arrows indicate aquaporin monomers, whereas arrowheads indicate PTMs and/or oligomerization of the channels. Molecular mass markers (kDa) are on the left.

The seabream rectal epithelium is mainly composed of elongated enterocytes, with microvilli in their apical membrane facing the lumen and the nuclei located in the basal half of the cells, as well as of goblet cells scattered within the epithelium containing intracellular round vacuoles (Figure 1A). To initially determine which aquaporin paralogs are expressed in the seabream rectal mucosa, we carried out Western blots of mucosal total membrane protein extracts using seabream-specific, affinity-purified antibodies against Aqp0a, -1aa, -1ab1, -1ab2, -3a, -4a, -8bb, -10bb and -14 (Table 1). The immunoblots showed reactive bands for Aqp0a, -1aa, -1ab1, -1ab2, -3a, -10bb and -14, with approximately the same molecular mass as the predicted monomers (28.7, 27.1, 28.2, 27.9, 32.7, 31.6, and 29.3 kDa, respectively) (Figure 1B), while Aqp4a and -8bb polypeptides were not detected. The Aqp0a, -1aa, -1ab1, -3a and -10bb immunoblots showed additional reactive bands of higher molecular mass than the corresponding monomers, which ranged from ~50 to 90 kDa, ~40 to 60 kDa, ~50 kDa, ~35 to 40 kDa, and 80 to 150 kDa, respectively (Figure 1B). The specificity of the reactions was confirmed by the preincubation of the antisera with the corresponding immunizing peptides (Figure 1B), suggesting that the high-molecular-mass bands detected may correspond to PTMs and/or oligomerization of the channels. In contrast, the Aqp1ab2 and -14 polypeptides were apparently not modified (Figure 1B).

**Figure 2:**
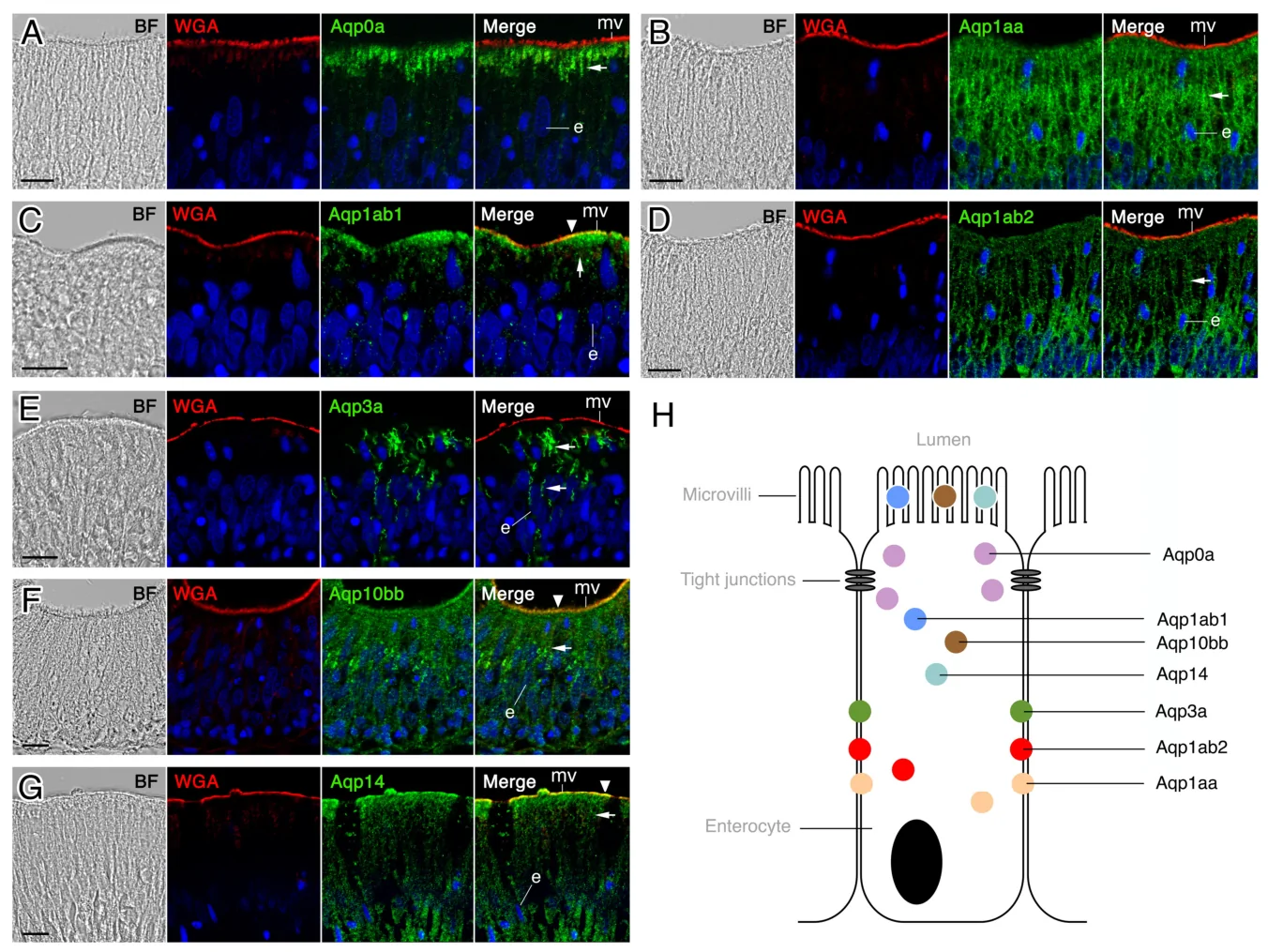
Differential immunolocalization of aquaporins in seabream rectal epithelia. (**A**–**G**) Representative brightfield (**left panels**) and immunofluorescence microscopy images of Aqp0a (**A**), Aqp1aa (**B**), Aqp1ab1 (**C**), Aqp1ab2 (**D**), Aqp3a (**E**), Aqp10bb (**F**) and Aqp14 (**G**) in enterocytes, using paralog-specific, affinity-purified rabbit or chicken polyclonal antibodies. The reactions were visualized with FITC-conjugated sheep anti-rabbit IgG or chicken IgY (green color), and the nuclei were counterstained with 4′,6-diamidino-2-phenylindole (DAPI; blue color). In all cases, the apical plasma membrane (microvilli) was labeled with Cy3-conjugated wheat germ agglutinin (WGA; red color). The arrows indicate the localization of the different channels in the cytoplasm, whereas the arrowheads indicate their location in the apical microvilli. e, enterocytes; mv, microvilli. Scale bars, 10 µm. Control sections incubated with antisera preadsorbed with the corresponding antigenic peptides were negative (see Figure S1). (**H**) Schematic diagram illustrating the subcellular distribution of the aquaporins investigated in the seabream enterocyte.

The cellular localization of the seven aquaporins in the seabream rectal epithelium was subsequently determined by immunofluorescence microscopy using affinity-purified antibodies and wheat germ agglutinin (WGA) as a marker of the enterocyte apical membrane. These experiments revealed that Aqp0a is localized in the subapical cytoplasm of the enterocytes (Figure 2A), while Aqp1aa remains cytoplasmatic and more accumulated in the basolateral membrane of the cells (Figure 2B). The duplicated Aqp1ab-type paralogs, Aqp1ab1 and -1ab2, show differential staining in enterocytes since Aqp1ab1 is localized in the subapical cytoplasm and apical plasma membrane (microvilli) (Figure 2C), whereas Aqp1ab2 is detected only spread in the cytoplasm with some more accumulation in the basolateral membrane of the cells, similar to Aqp1aa (Figure 2D). Aqp3a shows more restricted staining within the subapical cytoplasm as well as the basolateral membrane of the enterocytes, being the least widespread aquaporin of all the channels investigated (Figure 2E). In contrast, as observed for Aqp1ab1, Aqp10bb is dispersed in the cytoplasm and in the apical microvilli, also showing some discrete cytoplasmic accumulations in the middle region of the enterocytes (Figure 2F). Finally, as previously reported [16], Aqp14 is localized in the entire cytoplasm of the enterocytes as well as in the apical microvilli (Figure 2G). The specificity of the reactions for the different antibodies was confirmed with control sections that were incubated with antisera preabsorbed with the respective immunizing peptides (Figure S1). These data therefore indicate that multiple aquaporin paralogs display a complex pattern of subcellular localizations in seabream rectal enterocytes (Figure 2H), suggesting that alternative channel-mediated water and/or solute transport may be required for the proper physiological function of the rectum.

### 2.2. Short-Term Avp or Oxt Treatment in Vitro Differentially Regulates Apical Plasma Membrane Aquaporin Trafficking in Enterocytes

To investigate whether the neuropeptides Avp and Oxt can affect the intracellular trafficking of Aqp0a, -1aa, -1ab1, -1ab2, -3a, -10bb and -14, seabream rectal explants were treated ex vivo with controlled concentrations of the synthetic peptides Avp and Oxt (1 and 10 µM) for 3 h and subsequently analyzed by immunofluorescence microscopy followed by image analysis. Since seabream Avpr2aa and Oxtrb trigger distinct PKA and PKC signaling cascades [19], explants were treated with Avp in the presence or absence of the cAMP-dependent PKA inhibitor H89, whereas those treated with Oxt were exposed or not to the PKC inhibitor bisindolylmaleimide II (Bim II). In some experiments, to test whether the PKA or PKC signaling pathways could regulate aquaporin trafficking independently of the activation of the neuropeptide receptors, the explants were treated only with the cAMP-PKA activator forskolin (FSK) or the PKC agonist phorbol 12-myristate 13-acetate (PMA).

**Figure 3:**
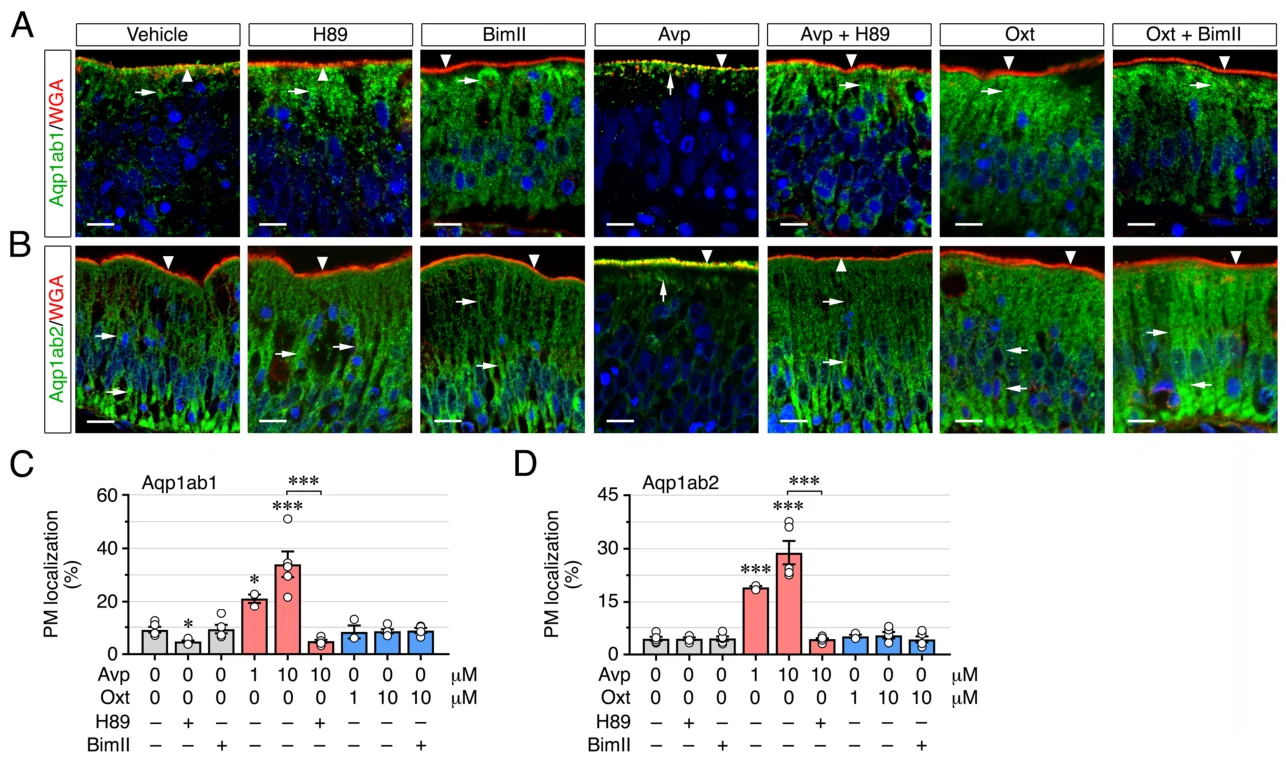
Avp regulates Aqp1ab1 and -1ab2 trafficking to the enterocyte plasma membrane. (**A**,**B**) Representative merged images of the immunostaining of Aqp1ab1 and -1ab2 ((**A**) and (**B**), respectively; green color) and WGA labeling (red color) in enterocytes from rectal explants exposed to Avp or Oxt, or vehicle (0.5% DMSO), in the presence or absence of 10 μM of PKA or PKC inhibitors (H89 and BimII, respectively), in vitro. The nuclei were counterstained with DAPI (blue color). The arrows indicate the localization of the different channels in the cytoplasm alone (Aqp1ab1) or also in the basolateral membrane (Aqp1ab2), whereas the arrowheads indicate their location in the apical microvilli. Scale bars: 10 µm. (**C**,**D**) Percentage (mean ± SEM; *n* = 3–5 different fish, white dots) of Aqp1ab1 (**C**) and Aqp1ab2 (**D**) in the enterocyte plasma membrane (PM) after each treatment, determined by image analysis. Data were statistically analyzed by one-way ANOVA. *, *p* < 0.05; ***, *p* < 0.001, with respect to the vehicle control group, or as indicated in brackets.

The results showed that neither Avp nor Oxt at the highest dose, nor PKA or PKC activation, affected the intracellular localization of Aqp0a, -1aa or -3a in the enterocytes, indicating that none of these signaling pathways seem to regulate the trafficking of these channels in the seabream rectal epithelium (Figure S2). In contrast, accumulation of both Aqp1ab1 and -1ab2 in the enterocyte apical microvilli increased after Avp treatment in a dose-dependent manner, by ~4- and ~6-fold, respectively, with 10 µM Avp compared to the control explants treated with vehicle (0.5% DMSO) (Figure 3). The Avp-induced apical plasma membrane targeting of Aqp1ab1 and -1ab2 was completely abolished in the presence of H89, suggesting the involvement of PKA signaling in the regulation of channel trafficking (Figure 3). Exposure of explants to H89 alone also decreased Aqp1ab1 abundance in the enterocyte apical microvilli by ~55% (Figure 3A,C), confirming the role of PKA in Aqp1ab1 trafficking. Conversely, the subcellular localization of Aqp1ab1 and -1ab2 in enterocytes was not affected by Oxt, regardless of the presence of BimII (Figure 3), which suggests that the Aqp1ab-type paralogs are not regulated by an oxytocinergic mechanism.

**Figure 4:**
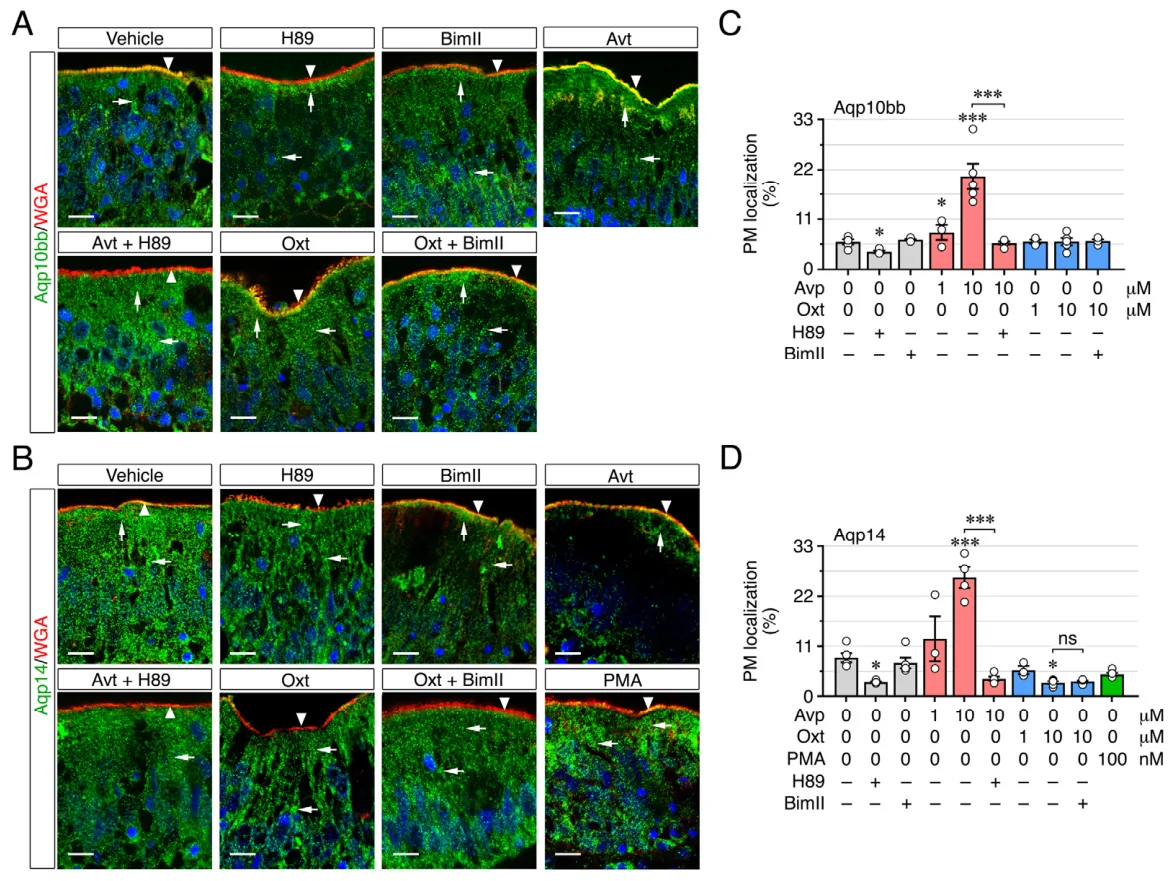
Avp and Oxt regulation of Aqp10bb and -14 trafficking in seabream enterocytes. (**A**,**B**) Representative merged images of the immunostaining of Aqp1ab1 and -1ab2 ((**A**) and (**B**), respectively; green color) and WGA labeling (red color) in enterocytes from rectal explants exposed to Avp or Oxt, or vehicle (0.5% DMSO), in the presence or absence of 10 μM of PKA or PKC inhibitors (H89 and BimII, respectively), in vitro. For Aqp14, the explants were also treated with 100 nM of the PKC activator PMA. The nuclei were counterstained with DAPI (blue color). The arrows indicate the localization of the different channels in the cytoplasm, whereas the arrowheads indicate their location in the apical microvilli. Scale bars: 10 µm. (**C**,**D**) Percentage (mean ± SEM; *n* = 3–5 different fish, white dots) of Aqp10bb (**C**) and Aqp14 (**D**) in the enterocyte plasma membrane (PM) after each treatment, determined by image analysis. Data were statistically analyzed by one-way ANOVA. *, *p* < 0.05; ***, *p* < 0.001, with respect to the vehicle control group, or as indicated in brackets. ns, not statistically significant.

As observed for Aqp1ab1 and -1ab2, apical plasma membrane targeting of Aqp10bb and -14 in rectal enterocytes was increased after vasopressinergic activation in a dose-dependent manner, although the accumulation of Aqp14 in the microvilli was only statistically significant in response to 10 µM Avp (Figure 4). Thus, the increase in channel abundance in the apical microvilli was ~3-fold for Aqp10bb and -14 (10 µM Avp) with respect to the controls, which was prevented in both cases in the presence of H89 (Figure 4). Aqp10bb and -14 accumulation in the apical membrane also decreased in the presence of H89 alone (Figure 4). These data therefore suggest the additional role of the Avp/PKA signaling pathway in regulating the trafficking of these channels. Treatment of explants with Oxt, in the presence or absence of BimII, had no effect on the trafficking of Aqp10bb (Figure 4A,C); however, the neuropeptide decreased the accumulation of Aqp14 by ~50% in the apical microvilli (Figure 4B,D). Such Oxt-triggered internalization of Aqp14 was not inhibited by BimII, but was partially mimicked by PMA (Figure 4B,D). These observations suggest that the internalization of Aqp14 in rectal enterocytes in response to oxytocinergic activation may be partially mediated by PKC and possibly by other kinases.

### 2.3. PKA Triggers Aqp1ab1, -1ab2, -10bb and -14 Phosphorylation and Apical Plasma Membrane Targeting in Enterocytes

**Figure 5:**
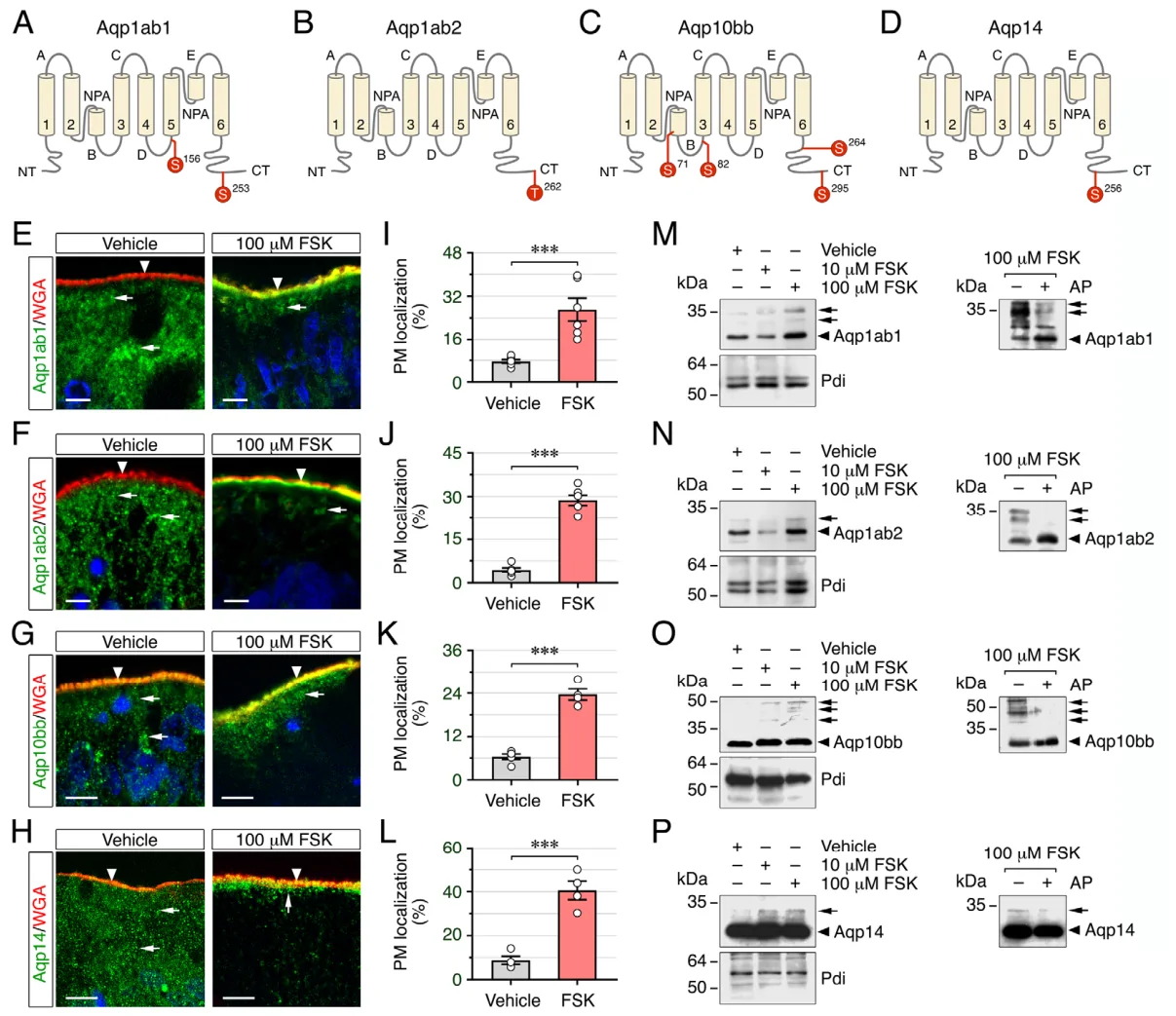
PKA activation in enterocytes enhances phosphorylation of Aqp1ab1, -1ab2, -10bb and -14 and apical plasma membrane targeting. (**A**–**D**) Schematic representation of seabream Aqp1ab1, -1ab2, -10bb and -14 monomers showing the six transmembrane domains (1–6), the conserved asparagine-proline-alanine (NPA) motifs, and the five connecting loops (**A**–**E**). For Aqp1ab1, -1ab2, and -14, the PKA phosphorylation sites involved in channel trafficking previously identified [16,18,19] are indicated. For Aqp10bb, four potential sites for PKA phosphorylation were identified after in silico prediction. (**E**–**H**) Representative merged images of Aqp1ab1 (**E**), -1ab2 (**F**), -10bb (**G**) and -14 (**H**) immunostaining (green color) and WGA labeling (red color) in enterocytes from rectal explants exposed to 100 μM of the PKA activator FSK or vehicle (0.5% DMSO) in vitro. The nuclei were counterstained with DAPI (blue color). The arrows indicate the localization of the different channels in the cytoplasm, whereas the arrowheads indicate their location in the apical microvilli. Scale bars, 10 µm. (**I**–**L**) Quantitative estimation (mean ± SEM, *n* = 4–6 different fish, white dots) of the amount of Aqp1ab1 (**I**), -1ab2 (**J**), -10bb (**K**) and -14 (**L**) in the apical plasma membrane (PM) of enterocytes in response to FSK, determined by image analysis. Data were statistically analyzed by the unpaired Student’s *t*-test (***, *p* < 0.001; as indicated in brackets). (**M**–**P**) Immunoblot of Aqp1ab1 (**M**), -1ab2 (**N**), -10bb (**O**) and -14 (**P**) in total membrane protein extracts from enterocytes isolated from rectal explants treated with 10 or 100 µM FSK, showing that the channels become phosphorylated after FSK treatment. Phosphorylated and non-phosphorylated aquaporin monomers are indicated by arrows and arrowheads, respectively. The protein disulfide isomerase (Pdi) is used as a loading control. Molecular mass markers (kDa) are indicated on the left. The (**right**) panels in (**M**–**P**) show the dephosphorylation of Aqp1ab1, -1ab2, -10bb and -14 in FSK (100 µM)-exposed enterocytes by alkaline phosphatase (AP) treatment. Phosphorylated and non-phosphorylated forms of the channels are indicated as above.

Previous mutagenesis studies using the *Xenopus laevis* oocyte expression system have identified Ser^156^ and Ser^253^ (intracellular loop D and C-terminus, respectively) in Aqp1ab1 (Figure 5A), Thr^262^ (C-terminus) in Aqp1ab2 (Figure 5B), and Ser^256^ (C-terminus) in Aqp14 (Figure 5D) as the PKA phosphorylation sites involved in channel trafficking in response to Avp [16,18]. In silico searches for putative PKA-mediated phosphorylation sites in Aqp10bb revealed Ser^71^ and Ser^82^, both in the intracellular loop B, and Ser^264^ and Ser^295^ in the C-terminus, as predicted target residues (Figure 5C). This is consistent with the Avp/PKA-mediated trafficking regulation of this channel, as suggested by the previous experiments. To corroborate that direct pharmacological activation of PKA can drive targeting of Aqp1ab1, -1ab2, -10bb and -14 to the enterocyte apical microvilli, rectal explants were treated with 100 µM of the PKA activator FSK, and the intracellular localization of the channels and their phosphorylation state were determined by immunofluorescence microscopy and immunoblotting, respectively.

Immunofluorescence microscopy showed that apical membrane trafficking of Aqp1ab1, -1ab2, -10bb and -14 in enterocytes was enhanced after FSK treatment (Figure 5E–H), by 3.5-, 6.5-, 3.7- and 4.6-fold, respectively (Figure 5I–L). Further immunoblotting analysis of FSK-exposed rectal explants revealed reactive protein bands of higher molecular masses than the corresponding monomers for the four channels, the intensity of which increased in a FSK dose-response manner (Figure 5M–P). To corroborate that these high molecular mass bands correspond to phosphorylated forms of the channels, protein extracts from explants treated with FSK were incubated with AP prior to immunoblotting analysis. The results showed that for Aqp1ab2, -10bb and -14, the upper bands disappeared after AP treatment (Figure 5N–P, right panels), indicating the FSK-mediated phosphorylation of the channels. In contrast, the Aqp1ab1 upper bands were only partially decreased in the presence of AP (Figure 5M, right panel), which suggests that additional posttranslational modifications of this channel, such as glycosylation, may occur in enterocytes. These data therefore suggest that Avp/PKA-mediated trafficking of Aqp1ab1, -1ab2, -10bb and -14 to the enterocyte apical microvilli is associated with the increased phosphorylation of the channels.

### 2.4. PKA-Mediated Aqp1ab1 and -1ab2 Trafficking in Enterocytes Is Regulated by 14-3-3ζ-like Protein Interaction

In seabream oocytes, Avp-mediated intracellular trafficking of Aqp1ab1 and -1ab2 to the microvilli during meiosis resumption and hydration involves the interaction of the channels with teleost-specific 14-3-3ζ-like binding proteins [18,19]. To investigate whether a similar mechanism also occurs during neurohormone regulation of Aqp1ab1 and -1ab2 trafficking in the enterocytes, the interaction of these channels with 14-3-3ζ-La and -Lb was examined in FSK-treated rectal explants using double-immunofluorescence microscopy, co-immunoprecipitation and immunoblotting.

**Figure 6:**
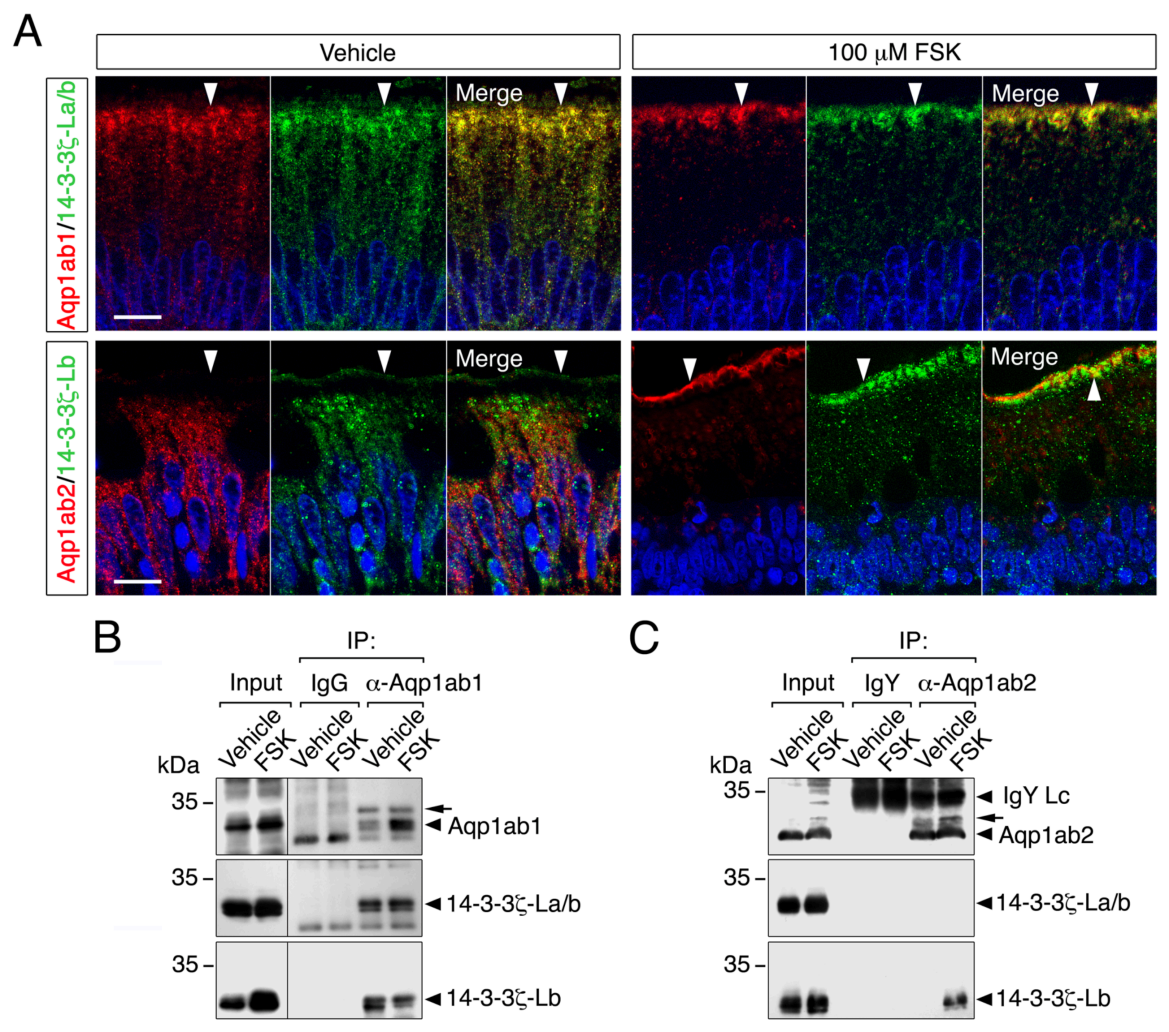
Aqp1ab1 and -1ab2 plasma membrane trafficking in enterocytes is regulated by PKA and 14-3-3ζ-like interaction. (**A**) Double immunostaining of Aqp1ab1 (red) and 14-3-3ζ-La/b (green), and Aqp1ab2 (red) and 14-3-3ζ-Lb (green) after FSK (100 µM) treatment. The nuclei were counterstained with DAPI (blue color). The arrowheads indicate the apical microvilli. Scale bars; 10 µm. (**B**,**C**) Co-immunoprecipitation of Aqp1ab1 (**B**) or -1ab2 (**C**) and 14-3-3ζ-La and 14-3-3ζ-Lb in enterocytes treated with vehicle or 100 µM FSK. The immunoprecipitated products (IP) using seabream Aqp1ab1 and -1ab2 specific antibodies were immunoblotted with appropriate antibodies (as indicated). IgG or IgY, immunoglobulin G or Y; IgG Lc, IgG light chain. Phosphorylated forms of the channels are indicated by arrows. In B-D, molecular mass markers (kDa) are indicated on the left.

In vehicle-treated explants, the immunostaining experiments showed the co-localization of Aqp1ab1 and 14-3-3ζ-La and -Lb in the cytoplasm as well as in the subapical region of the enterocytes, while exposure to FSK visibly promoted the trafficking of Aqp1ab1/14-3-3ζ-La/b complexes to the enterocyte apical membrane (Figure 6A). The co-immunoprecipitation assays indicated that Aqp1ab1 was already phosphorylated and bound to 14-3-3ζ-La and -Lb in the absence of FSK (Figure 6B). However, after FSK treatment, the channel became somewhat more phosphorylated, while maintaining the interaction with both 14-3-3ζ-L proteins (Figure 6B), which coincided with the trafficking of the channel to the enterocyte microvilli. Phosphorylation of Aqp1ab2 was also increased after FSK stimulation, but in this case, the channel only interacts with 14-3-3ζ-Lb, and specifically when it is translocated from the basolateral membrane to the enterocyte apical membrane (Figure 6A,C). These observations suggest that, as in oocytes, the Avp/PKA transduction pathway regulates Aqp1ab1 and -1ab2 trafficking in rectal enterocytes through differential interaction with 14-3-3ζ-La and -Lb binding proteins.

## 3. Discussion

From a historical perspective, the vasopressinergic regulation of facilitated water uptake was long suspected based on the effects of neurohypophysial pituitrin injections in frogs [10,40]. It was eventually demonstrated in mammals soon after the discovery of aquaporins [41,42]. Subsequent studies in lungfishes, amphibians and birds revealed that such neuropeptide signaling pathways are also involved in their homeosmotic regulation under desiccating conditions [43,44,45]. However, in teleosts, including the gilthead seabream, despite corroborating evidence suggesting that such mechanisms are associated with their hypoosmotic regulation in the desiccating environment of seawater [30,32,46], the signal transduction pathways linking Avp and Oxt to intestinal water absorption have remained poorly known. In this study, we show that multiple water channel paralogs, including the classical aquaporins Aqp0a, -1aa, -1ab1, -1ab2, and -14, and the aquaglyceroporins Aqp3a and -10bb, are localized in the rectal enterocytes of gilthead seabream.

Our observation that the classical aquaporins Aqp1aa and -1ab-type channels and the aquaglyceroporins Aqp3a and -10bb display intestinal expression is consistent with previous studies in marine teleosts ranging from elopomorph eels [1,28,47,48,49] to modern acanthomorph species [16,24,27,32,50,51,52]. This may suggest that their recruitment to the intestinal water absorption pathway is conserved in marine teleosts. In contrast to some eels and salmonids [25,49,53,54,55], however, we did not currently detect expression of Aqp8-type paralogs in seabream enterocytes. Such disparities could reflect species-specific differences or the absence of sufficient cross-reaction between our Aqp8bb antibody and the Aqp8aa and -8ab paralogs also encoded in the seabream genome. Similarly, the absence of intestinal Aqp4-type channel expression in seabream contrasts with that of mammals, in which AQP4 is localized in the colonic surface epithelium [56], but is consistent with earlier studies which report that orthologous Aqp4 expression appears to be very low or absent in both the dogfish shark [57] and marine teleosts [28,58]. Conversely, as shown here and previously [16], the Aqp4-related Aqp14 channel is highly expressed in seabream rectal enterocytes.

The rectal localization of Aqp0a has not previously been noted in marine teleosts. In mammals, it is well known that AQP0 is essential for lens transparency, homeostasis and cell-to-cell adhesion [59,60]. This is also the case in teleosts, where two paralogs (Aqp0a and -b) exist in diploid species [61], whereas in paleotetraploid Atlantic salmon (*Salmo salar*), which encodes tetraparalogous *aqp0a1*, *-a2*, *-0b1* and *-0b2* genes, ~1000-fold lower mRNA expression was recorded in the rectum compared to the lens [62]. In the present context, the localization of Aqp0a in the subapical cytoplasm of the enterocytes, possibly near tight junctions, might suggest a dual function of the protein. The first role potentially arises from the fact that teleost Aqp0 channels retain cell-cell adhesion properties in addition to water permeability [62,63]. Consequently, the role of Aqp0a at the tight junctions between enterocytes may in part be for structural purposes, similar to claudins or occludins in the corneal endothelium [28], while the second role could facilitate paracellular water transport. Such possibilities would need to be tested in the future.

In addition to their localization in the posterior and rectal intestine, the functional basis of aquaporins contributing to water absorption in marine teleosts also partially arises from data showing that all of the currently investigated paralogs are permeable to water, or, in the case of the aquaglyceroporins, to water, urea and glycerol [16,18,38,39]. More compelling evidence arises from our explant in vitro experiments demonstrating that four of the paralogs (Aqp1ab1, -1ab2, -14 and -10bb) traffic to the apical plasma membranes of the microvilli in response to the vasopressinergic induction of the Avpr2aa-PKA signaling pathway. These data agree with previous ex vivo studies of seabream oocytes, where Aqp1ab1 and -1ab2 trafficking to the plasma membrane is differentially regulated through the Avp-Avpr2aa-PKA axis [18,19]. A similar regulation is observed for Aqp14 expressed in *X. laevis* oocytes, where Avp-activated PKA increases the oocyte osmotic water permeability and PKC activated by Avpr1 or Oxtr receptors inhibits the function of the channel [16]. In the present study, we confirmed the oxytocinergic regulation of Aqp14 recycling from the apical microvillar membranes of seabream enterocytes, but unlike the PKC-mediated route in frog oocytes, the signaling cascade likely also involves other kinases. In contrast, the enterocyte trafficking of Aqp0a, -1aa and -3a was not affected by Avp or Oxt, suggesting that these channels may be regulated by other endocrine pathways.

In seabream oocytes, C-terminal phosphorylation of the Aqp1ab-type paralogs primes the binding of 14-3-3ζ-La and -Lb proteins to enhance membrane trafficking, while a second phosphorylation in Aqp1ab1 loop D completes the translocation to the apical membrane microvilli [18,19]. The co-immunoprecipitation experiments using FSK-treated explants suggest that a similar mechanism occurs during the Avpr2aa-PKA-induced apical membrane trafficking of Aqp1ab1 and -1ab2 in enterocytes. However, whether the channels are differentially localized within the proximal and distal regions of the enterocyte microvilli, as in oocytes [18,19], is yet to be determined.

**Figure 7:**
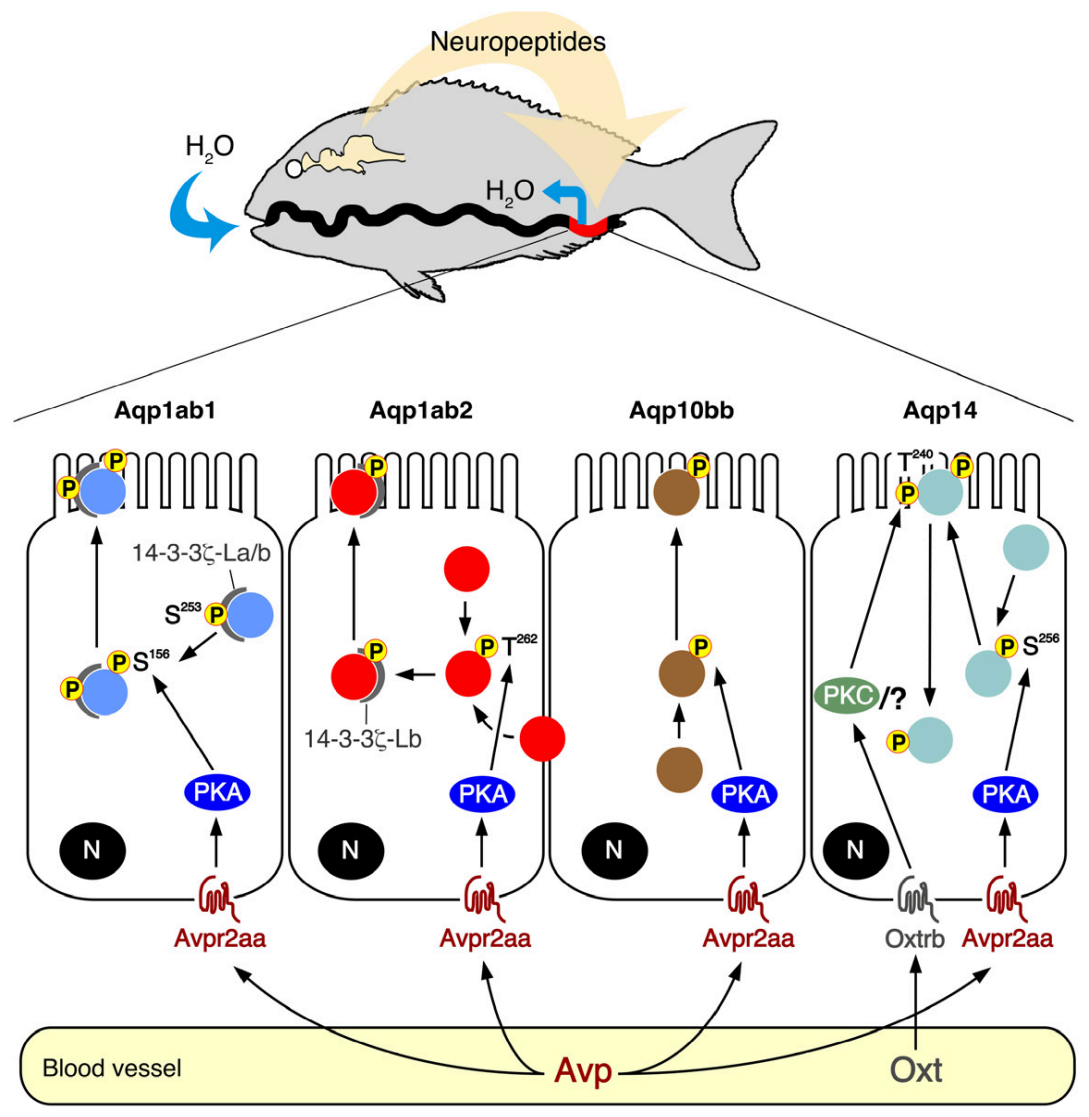
Proposed model for neurohormone regulation of aquaporin trafficking in seabream rectal enterocytes. Following phosphorylation (P) of Aqp1ab1 C-terminal Ser^253^ and binding of 14-3-3ζ-La and -Lb, the release of the neuropeptide Avp, possibly through activation of the Avpr2aa, drives PKA-mediated phosphorylation of Aqp1ab1 loop D Ser^156^, targeting the channel to the apical microvilli. Avp-stimulated Avpr2aa also triggers PKA-mediated phosphorylation of C-terminal Thr^262^ in Aqp1ab2, promoting the binding to 14-3-3ζ-Lb and the trafficking of the channel from the cytoplasm and basolateral membrane to the apical plasma membrane. Similarly, Avp seems to trigger the phosphorylation of Aqp10bb and apical membrane trafficking of the channel, although in this case, it is not known if binding to 14-3-3 proteins is necessary. Finally, Aqp14 is under dual regulation, where Avp/PKA signaling triggers C-terminal Ser^256^ phosphorylation and apical microvilli targeting, while the activation of the Oxtrb in enterocytes drives C-terminal Thr^240^ phosphorylation through PKC and/or another yet unknown kinase and channel internalization.

Combining the present data with previous mutagenesis studies on seabream aquaporin paralogs [16,18,19], we propose an integrated model for the neurohormone regulation of aquaporin trafficking in seabream rectal enterocytes (Figure 7). In this model, circulating Avp produced in the neurohypophysis binds to the Avpr2aa receptor located in the basolateral membrane of the enterocytes to activate PKA-mediated phosphorylation of either loop D or C-terminal residues to promote apical membrane trafficking of the intracellularly localized aquaporins. In the case of Aqp1ab1, which is already bound to 14-3-3ζ-La and -Lb proteins following the phosphorylation of C-terminal Ser^253^, PKA-mediated phosphorylation of loop D Ser^156^ initiates the trafficking. For Aqp1ab2, the Avp-Avpr2aa-PKA pathway induces the phosphorylation of C-terminal Thr^262^, resulting in the preferential binding of 14-3-3ζ-Lb and trafficking from the basolateral membrane of the enterocytes to the apical membrane. Conversely, for Aqp10bb and -14, apical membrane targeting is activated through the Avp-Avpr2aa-PKA cascade to phosphorylate an as yet unknown residue in Aqp10bb or Ser^256^ in Aqp14. Finally, recycling of Aqp14 is initiated via the Oxt-Oxtrb pathway to activate PKC and possibly other yet unknown kinases, which phosphorylate the channel C-terminal Thr^240^. This model implies that a common vasotocinergic pathway regulates the apical membrane insertion of multiple aquaporin paralogs, possibly for luminal water entry into the rectal enterocytes. The seabream rectal enterocytes also express Aqp1aa and -3a in the basolateral membranes, as has been reported for Aqp1aa, -8ab and -8bb paralogs in the mid- and posterior enterocytes and the kidney proximal tubules of salmonids [25,64]. It is thus possible that Aqp1aa and -3a may complete a serosal-directed transcellular water transport via the paracellular route enabled by active NaCl absorption generated by colocalized ion pumps and passive channels [2,65,66].

In summary, this work suggests that multiple aquaporins, including four classical channels and two aquaglyceroporins, are possibly involved in the post-imbibed absorption of water and solutes by seabream rectal enterocytes. Since Aqp1-type, Aqp3-type and Aqp10-type channels are also intestinally expressed in other species ranging from elopomorph eels to modern acanthomorph fishes, it seems possible that the water absorptive role of these channels may be conserved in marine teleosts. Vasopressinergic regulation via the Avp-Avpr2aa-PKA axis induces the trafficking of Aqp1ab1, -1ab2, -10bb and -14 to the apical microvilli plasma membranes, with the Aqp1ab-type membrane targeting enhanced via phosphorylation priming and the binding of 14-3-3ζ-like carrier proteins. Oxytocinergic regulation seems to be important for Aqp14 recycling, suggesting that this latter channel may act as an osmosensor. A transcellular pathway for water absorption can be envisaged via luminal uptake across the apical membrane microvilli and paracellular exit to the serosal side via Aqp1aa or -3a. Whether other aquaporins not investigated in the present study may also facilitate a transcellular or paracellular pathway remains to be determined. The combined data nevertheless suggest that as in sarcopterygians, marine gilthead seabream water-conservation is in part regulated by the same neuropeptide signaling pathways through aquaporin intracellular trafficking in rectal enterocytes.

## 4. Materials and Methods

### 4.1. Experimental Animals and Tissue Collection

Adult male seabream (~1 kg, 2 years old) were maintained in 2500-L tanks under natural conditions of salinity (~35‰), temperature and photoperiod at the facilities of the Institute of Marine Sciences, Spanish National Research Council (CSIC, Madrid, Spain). Prior to tissue collection, fish were fasted for 4 days to minimize the presence of feces in the gut. The fish were euthanized with 2000 ppm phenoxyethanol, and biopsies from the rectum were excised and rinsed in phosphate-buffered saline (PBS). Samples were immediately processed for histology and immunofluorescence microscopy or transferred to L-15 Leibovitz culture medium without phenol red (Gibco, Grand Island, NY, USA, #21083027) for further in vitro incubations. In some experiments, the mucosa containing the epithelial cells was scraped from the rectal biopsies using a sterile blade and subsequently stored at -80 °C for further protein extraction. Procedures related to the care and use of fish and sample collection were approved by the Ethics Committee of CSIC and the Department of Climate Action, Food and Rural Agenda, General Directorate of Environmental Policies and the Natural Environment (Government of Catalonia) (project no. 12147).

### 4.2. Aquaporin Sequence Analysis

In silico searches for potential phosphorylation sites in the seabream Aqp10bb amino acid sequence (GenBank accession no. AAR13054) were carried out using the NetPhos 3.1 Server (http://www.cbs.dtu.dk/services/NetPhos/, accessed on 1 March 2024) [67] and The Eukaryotic Linear Motif resource (http://elm.eu.org/, accessed on 1 March 2024) [68].

### 4.3. Histology

Pieces of the rectum were fixed in Bouin’s fluid (75 mL picric acid in 95% ethanol, 25 mL 37–40% formaldehyde and 5 mL glacial acetic acid) for 16 h at room temperature, dehydrated, and embedded in paraffin (Paraplast, Merck, Darmstadt, Germany, #P3558). Sections of 7 μm in thickness were obtained with a Leica rotary microtome (Leica RM 2255, Leica Biosystems, Wetzlar, Germany), stained with hematoxylin-eosin, and finally mounted with Eukitt Quick-hardening mounting medium (Merck, #03989) for examination using a Nikon Eclipse 90i light microscope (Tokyo, Japan).

### 4.4. Incubation of Rectal Explants in Vitro

Rectal biopsies were sliced into small pieces of ~0.5 cm and placed into two 12-well culture plates containing 1 mL of L-15 Leibovitz medium supplemented with gentamycin (100 μg/ml) and 10 mM Hepes, at pH 7.5. For each fish, the explants were treated in duplicate with 1 or 10 μM of synthetic Avp (CYIQNCPRG-NH2) or Oxt (CYISNCPIG-NH2) [66] (both purchased from PeptideSynthetics, Peptide Protein Research Ltd., Hampshire, UK), or the hormone vehicle (0.5% DMSO, control), in the presence or absence of 10 μM of the PKA or PKC inhibitors, H89 and Bim II, respectively. The inhibitors were added 1 h prior to the treatment with Avp or Oxt. In other experiments, the explants were treated with 100 nM of the PKC activator PMA or preincubated with 100 μM of the phosphodiesterase inhibitor 3-isobutyl-1-methylxanthine (IBMX) for 1 h and subsequently exposed to 100 μM of FSK. In all experiments, the explants were incubated for up to 3 h in a temperature-controlled incubator at 18 °C. Intact explants were then processed for immunofluorescence microscopy, while the mucosa from duplicated explants was collected as described above for further immunoblotting.

### 4.5. Immunofluorescence Microscopy

The rectal samples were fixed in 4% paraformaldehyde (PFA) in PBS for 6 h at room temperature and dehydrated and embedded in paraffin, as described above. Sections were attached to UltraStick/UltraFrost Adhesion Slides (Electron Microscopy Science, Morgantown, PA, USA, #3039) and rehydrated in decreasing xylene and ethanol solutions. The sections were permeabilized with 0.2% Triton X-100 in PBS for 10 min before blocking with PBS containing 5% normal goat serum and 0.1% BSA for 1 h at room temperature. Immunofluorescence was carried out using custom-made seabream-specific aquaporin antibodies (Table 1) or commercial rabbit antibodies against human 14-3-3ζ proteins, which recognize both seabream 14-3-3ζ-La and -Lb orthologs (GeneTex, Irvine, CA, USA, #GTX101075) or only the 14-3-3ζ-Lb paralog (Signalway Antibody, Greenbelt, MD, USA, #SAB40525) [18,19]. In some experiments, a mouse monoclonal antibody against Na^+^-K^+^ ATPase (ATP1A1 antibody a5, Developmental Study Hybridoma Bank, University of Iowa, Iowa City, IA, USA) was also employed. Sections were incubated with the primary antibodies diluted 1:500 in PBS overnight at 4 °C. Antigen–antibody reactions were revealed with Cy3-conjugated sheep anti-rabbit IgG antibody (Merck, #C2306), Alexa Fluor 488-conjugated goat anti-chicken IgY antibody (Invitrogen, Carlsbad, CA, USA #A-11039) or Alexa Fluor 488 goat anti-mouse IgG (Invitrogen, #A-11001), at a 1:1000 dilution for 1 h at room temperature. The sections were counterstained with Alexa 647-coupled wheat germ agglutinin (WGA) diluted in PBS (1:5000) for 10 min to stain membranes, whereas the nuclei were counterstained with 4′,6-diamidino-2-phenylindole (DAPI) (1:10,000 diluted in PBS) for 3 min. Control sections were incubated with aquaporin antibodies preadsorbed with the corresponding peptides used for immunization or with secondary antibodies only. For double labeling of Aqp1ab1 and 14-3-3ζ with rabbit-derived antibodies, 14-3-3ζ was detected first with a Cy3-conjugated anti-rabbit secondary antibody. The sections were then fixed for 1 h in 4% PFA before a 2 h incubation with Aqp1ab1 antibody directly labeled using the Zenon Rabbit IgG Alexa Fluor 488 Labeling Kit (Invitrogen, #Z25302), in accordance with the manufacturer’s protocol. In all cases, the sections were washed and mounted with fluoromount aqueous antifading medium (Merck, #F4680) and examined and photographed with a Zeiss Axio Imager Z1/ApoTome fluorescence microscope (Carl Zeiss, Jena, Germany).

### 4.6. Image Processing and Analysis

Apical enrichment of aquaporin immunostaining was quantified using the ImageJ v.1.52o software (https://imagej.nih.gov/ij/, accessed on 1 November 2024). For each fish, six to eight micrographs representing duplicate treatments were analyzed. WGA labeling delineated the apical microvillar membrane, for which a 0.5 µm^2^ region was selected; a 15 µm^2^ region represented the enterocyte cytoplasm. The mean fluorescence intensity in each region was multiplied by its area. Apical fluorescence was then calculated as a percentage of the combined fluorescence from the apical and cytoplasmic regions. Images from all experimental groups were recorded with matched exposure settings and comparable orientations, maintaining an identical combined sampling area.

### 4.7. Protein Extraction from the Rectal Mucosa

Total protein fractions of the mucosa were isolated by homogenization of the frozen cells (~100 mg) with 1.5 mL of RIPA lysis buffer (300 mM NaCl, 100 mM Tris-HCl pH 8, 2% Triton X-100, 1% sodium deoxycholate, 2 mM EDTA, 2 mM EGTA, and EDTA-free protease inhibitors), supplemented with 200 U of benzonase and 2 mM of the phosphatase inhibitors Na_3_VO_4_ and NaF, in a 2 mL glass douncer on ice. The samples were transferred to 1.5 mL Eppendorf tubes and centrifuged at 10,000× *g* for 1 min at 4 °C. The supernatant (300 µL) was recovered and mixed with 100 µL of 4 × Laemmli sample buffer plus 5% β-mercaptoethanol, denatured at 90 °C for 15 min, flash-frozen in liquid nitrogen and stored at −80 °C until immunoblotting. The total membrane fraction was isolated by homogenization of the cells with 1 mL of HbA buffer (20 mM Tris, pH 7.4, 5 mM MgCl_2_, 5 mM NaH_2_PO_4_, 1 mM EDTA, 80 mM sucrose, and EDTA-free protease inhibitors) [69], followed by centrifugation at 200× *g* for 5 min at 4 °C. The supernatant was transferred to a new 1.5 mL tube and centrifuged again. The resulting supernatant was centrifuged at 20,000× *g* for 20 min at 4 °C, and the pellet was resuspended in 100 µL of 1 × Laemmli or, in some cases, in 100 µL of 4 × Laemmli containing 8 M urea-2M thiourea and 5% β-mercaptoethanol (β-ME). Both solutions were supplemented with 1 mM of phosphatase and protease inhibitors. The urea-containing extracts were heated at 42 °C for 10 min, while the other samples were denatured at 90 °C for 10 min, before proceeding with immunoblotting.

### 4.8. Western Blot

Protein extracts (20–30 mg) were subjected to 12% sodium dodecyl sulfate polyacrylamide gel electrophoresis (SDS-PAGE), as described previously [38]. To determine whether some aquaporins were phosphorylated, samples were treated or not with 10 U/µg calf intestine alkaline phosphatase (AP) (New England Biolabs Inc., Ipswich, MA, USA, #M0525) for 1 h at 37 °C before electrophoresis. Following electrophoresis, proteins were transferred to 0.2 μm Immun-Blot nitrocellulose membranes (Bio-Rad Laboratories, Inc., Hercules, CA, USA, #1620112). Nonspecific binding was blocked for 1 h with 5% nonfat dry milk in TBST (20 mM Tris, 140 mM NaCl, 0.1% Tween, pH 7.6). The membranes were incubated with primary antibodies diluted in blocking solution (from 1:300 to 1:1000) overnight at 4 °C. As loading controls, a monoclonal antibody against α-tubulin (Merck, #T9026) or a rabbit anti-protein disulfide isomerase (PDI) antibody produced in rabbit (Merck, #P7496) were also used at a 1:25,000 and 1:2000 dilution, respectively. Horseradish peroxidase-conjugated anti-rabbit IgG (BioRad, Hercules, CA, USA, #172-1019) and anti-chicken IgY (Thermo-Fisher, Waltham, MA, USA, #A16054), each diluted 1:5000, were used to detect bound primary antibodies. Signals were developed with ImmobilonTM Western Chemiluminescent HRP Substrate (Merck, #WBKLS) and recorded on X-ray film. The specificity of the reactions was assessed by preabsorbing the antibodies with their corresponding peptides (1:10 ratio) for 1 h at 37 °C prior to dilution in milk and incubation with the membrane.

### 4.9. Co-Immunoprecipitation

Co-immunoprecipitation was performed on frozen rectal mucosa biopsies using the Pierce™ Crosslink Magnetic IP/Co-IP Kit (Pierce, IL, USA, #88805). Tissue was disrupted on ice with a glass douncer in the supplied lysis buffer, followed by centrifugation for 1 min at 14,000× *g*. Ten percent of each lysate was retained as the input fraction and combined with 4 × Laemmli sample buffer containing 5% β-ME. The remaining lysate was mixed with magnetic beads coupled to rabbit anti-seabream Aqp1ab1 antibody and agitated overnight at 4 °C. Bound proteins were recovered in 50 µL of kit elution buffer and combined with 4 × Laemmli buffer containing 5% β-ME and protease and phosphatase inhibitors. For Aqp1ab2 precipitation, chicken anti-Aqp1ab2 antibody was coupled to Dynabeads™ M-280 Tosylactivated (Invitrogen, #14203) according to the supplier’s protocol. Parallel precipitations with rabbit IgG or chicken IgY served as negative controls. Input and recovered fractions were examined by SDS-PAGE and immunoblotting using the procedures described above.

### 4.10. Statistical Analysis

All statistical calculations were performed in GraphPad Prism v9.1.2. For comparisons involving two independent groups, an unpaired two-tailed Student’s *t*-test was applied, or a Mann–Whitney test when group variances were unequal. Analyses involving more than two groups used one-way ANOVA with Tukey’s post hoc comparisons or Kruskal–Wallis analysis followed by Dunn’s test for nonparametric comparisons. Percentage data were square-root-transformed before testing. Significance thresholds were *p* < 0.05 (*), *p* < 0.01 (**), and *p* < 0.001 (***).

## Figures and Tables

**Table 1:** Seabream-specific aquaporin antibodies used in the present study.

Aquaporin Paralog	GenBank acc. No.	Antigenic Peptide Sequence ^1^	Host	Reference
Aqp0a	AGT57405	AEGQQETRGEPIELKTQAL	Rabbit	[38]
Aqp1aa	AAV34610	PKFDDFPERMKVLVS	Rabbit	[18]
Aqp1ab1	AAV34609	PREGNSSPGPSQGPSQWPKH	Rabbit	[18]
Aqp1ab2	MW960021	REPLLEDVKDRWSRT	Chicken	[18]
Aqp3a	KC788197	NVASNDNSLKATKEM	Chicken	[39]
Aqp4a	KY682700	SDPEKSEKKDLFQDSTGE	Chicken	[39]
Aqp8bb	DQ889225	LGDRKMRLILK	Rabbit	[38]
Aqp10bb	AAR13054	QEATEEKAGVELEGVK	Rabbit	[39]
Aqp14	MK883753	TQNAMRAKQANKQENN	Rabbit	[16]

^1^ All sequences are in the cytoplasmic C-terminus of the predicted proteins.

## Data Availability

All relevant data can be found within the article and its Supplementary Information.

## Supplementary Materials

The following supporting information can be downloaded at: https://www.mdpi.com/article/10.3390/ijms27219427/s1.

## References

[1] Takei Y. (2021). The digestive tract as an essential organ for water acquisition in marine teleosts: Lessons from euryhaline eels. Zool. Lett..

[2] Grosell M., Mager E.M., Williams C., Taylor J.R. (2009). High rates of HCO_3_^−^ secretion and Cl^–^ absorption against adverse gradients in the marine teleost intestine: The involvement of an electrogenic anion exchanger and H^+^-pump metabolon?. J. Exp. Biol..

[3] Evans D.H. (2008). Teleost fish osmoregulation: What have we learned since August Krogh, Homer Smith, and Ancel Keys. Am. J. Physiol. Regul. Integr. Comp. Physiol..

[4] Whittamore J.M. (2012). Osmoregulation and epithelial water transport: Lessons from the intestine of marine teleost fish. J. Comp. Physiol. B.

[5] Grosell M., Oehlert A.M. (2023). Staying hydrated in seawater. Physiology.

[6] Cerdà J., Finn R.N. (2010). Piscine aquaporins: An overview of recent advances. J. Exp. Zool. A Ecol. Genet. Physiol..

[7] Sundell K., Sundh H. (2012). Intestinal fluid absorption in anadromous salmonids: Importance of tight junctions and aquaporins. Front. Physiol..

[8] Breves J.P. (2020). Hormonal regulation of aquaporins in fishes. Vitam. Horm..

[9] Engel A., Walz T., Agre P. (1994). The aquaporin family of membrane water channels. Curr. Opin. Struct. Biol..

[10] Finn R.N., Cerdà J., Choi S. (2018). Aquaporin. Encyclopedia of Signaling Molecules.

[11] Kitchen P., Salman M.M., Pickel S.U., Jennings J., Törnroth-Horsefield S., Conner M.T., Bill R.M., Conner A.C. (2019). Water channel pore size determines exclusion properties but not solute selectivity. Sci. Rep..

[12] Bill R.M., Hedfalk K. (2021). Aquaporins—Expression, purification and characterization. Biochim. Biophys. Acta Biomembr..

[13] Finn R.N., Chauvigné F., Hlidberg J.B., Cutler C.P., Cerdà J. (2014). The lineage-specific evolution of aquaporin gene clusters facilitated tetrapod terrestrial adaptation. PLoS ONE.

[14] Finn R.N., Cerdà J. (2011). Aquaporin Evolution in Fishes. Front. Physiol..

[15] Finn R.N., Cerdà J. (2015). Evolution and functional diversity of aquaporins. Biol. Bull..

[16] Chauvigné F., Yilmaz O., Ferré A., Fjelldal P.G., Finn R.N., Cerdà J. (2019). The vertebrate Aqp14 water channel is a neuropeptide-regulated polytransporter. Commun. Biol..

[17] Yilmaz O., Chauvigné F., Ferré A., Nilsen F., Fjelldal P.G., Cerdà J., Finn R.N. (2020). Unravelling the complex duplication history of deuterostome glycerol transporters. Cells.

[18] Ferré A., Chauvigné F., Vlasova A., Norberg B., Bargelloni L., Guigó R., Finn R.N., Cerdà J. (2023). Functional evolution of clustered aquaporin genes reveals insights into the oceanic success of teleost eggs. Mol. Biol. Evol..

[19] Ferré A., Chauvigné F., Gozdowska M., Kulczykowska E., Finn R.N., Cerdà J. (2023). Neurohypophysial and paracrine vasopressinergic signaling regulates aquaporin trafficking to hydrate marine teleost oocytes. Front. Endocrinol..

[20] Finn R.N., Cerdà J. (2020). Genetic adaptations for the oceanic success of fish eggs. Trends Genet..

[21] Li S., Li C., Wang W. (2020). Molecular aspects of aquaporins. Vitam. Horm..

[22] Suzuki M. (2010). Expression and localization of aquaporin-1 on the apical membrane of enterocytes in the small intestine of bottlenose dolphins. J. Comp. Physiol. B.

[23] Zhu C., Chen Z., Jiang Z. (2016). Expression, distribution and role of aquaporin water channels in human and animal stomach and intestines. Int. J. Mol. Sci..

[24] Cao Q., Blondeau-Bidet E., Lorin-Nebel C. (2022). Intestinal osmoregulatory mechanisms differ in Mediterranean and Atlantic European sea bass: A focus on hypersalinity. Sci. Total Environ..

[25] Engelund M.B., Chauvigné F., Christensen B.M., Finn R.N., Cerdà J., Madsen S.S. (2013). Differential expression and novel permeability properties of three aquaporin 8 paralogs from seawater-challenged Atlantic salmon smolts. J. Exp. Biol..

[26] Madsen S.S., Olesen J.H., Bedal K., Engelund M.B., Santamaría Y.V., Tipsmark C.K. (2011). Functional characterization of water transport and cellular localization of three aquaporin paralogs in the salmonid intestine. Front. Physiol..

[27] Raldúa D., Otero D., Fabra M., Cerdà J. (2008). Differential localization and regulation of two aquaporin-1 homologs in the intestinal epithelia of the marine teleost *Sparus aurata*. Am. J. Physiol. Reg. Integr. Comp. Physiol..

[28] Madsen S.S., Engelund M.B., Cutler C.P. (2015). Water transport and functional dynamics of aquaporins in osmoregulatory organs of fishes. Biol. Bull..

[29] Cheng H.M., Mah K.K., Seluakumaran K., Cheng H.M., Mah K.K., Seluakumaran K. (2020). Intestinal fluid handling: Absorption. Defining Physiology: Principles, Themes, Concepts. Volume 2: Neurophysiology and Gastrointestinal Systems.

[30] Kulczykowska E., Baldisserotto B. (2007). Arginine vasotocin and isotocin: Towards their role in fish osmoregulation. Fish Osmoregulation.

[31] Hausmann H., Meyerhof W., Zwiers H., Lederis K., Richter D. (1995). Teleost isotocin receptor: Structure, functional expression, mRNA distribution and phylogeny. FEBS Lett..

[32] Martos-Sitcha J.A., Campinho M.A., Mancera J.M., Martínez-Rodríguez G., Fuentes J. (2015). Vasotocin and isotocin regulate aquaporin 1 function in the sea bream. J. Exp. Biol..

[33] Cao Q., Blondeau-Bidet E., Lorin-Nebel C. (2022). Hypo-osmoregulatory roles of vasotocinergic and isotocinergic systems in the intestines of two european sea bass lineages. Int. J. Mol. Sci..

[34] Cerdà J., Zapater C., Chauvigné F., Finn R.N. (2013). Water homeostasis in the fish oocyte: New insights into the role and molecular regulation of a teleost-specific aquaporin. Fish Physiol. Biochem..

[35] Chaube R., Chauvigné F., Tingaud-Sequeira A., Joy K., Acharjee A., Singh V., Cerdà J. (2010). Molecular and functional characterization of catfish (*Heteropneustes fossilis*) aquaporin-1b: Changes in expression during ovarian development and hormone-induced follicular maturation. Gen. Com. Endocrinol..

[36] Gregório S.F., Carvalho E.S., Encarnação S., Wilson J.M., Power D.M., Canário A.V., Fuentes J. (2013). Adaptation to different salinities exposes functional specialization in the intestine of the sea bream (*Sparus aurata* L.). J. Exp. Biol..

[37] Martos-Sitcha J.A., Gregório S.F., Carvalho E.S., Canario A.V., Power D.M., Mancera J.M., Martínez-Rodríguez G., Fuentes J. (2013). AVT is involved in the regulation of ion transport in the intestine of the sea bream (*Sparus aurata*). Gen. Comp. Endocrinol..

[38] Chauvigné F., Boj M., Vilella S., Finn R.N., Cerdà J. (2013). Subcellular localization of selectively permeable aquaporins in the male germ line of a marine teleost reveals spatial redistribution in activated spermatozoa. Biol. Reprod..

[39] Chauvigné F., Parhi J., Ducat C., Ollé J., Finn R.N., Cerdà J. (2018). The cellular localization and redistribution of multiple aquaporin paralogs in the spermatic duct epithelium of a maturing marine teleost. J. Anat..

[40] Brunn F. (1921). Beitrag zur kenntnis der wirkung von hypophysenertrakten auf den wasserhaushalt des frosches. Z. Ges. Exp. Med..

[41] Preston G.M., Carroll T.P., Guggino W.B., Agre P. (1992). Appearance of water channels in *Xenopus* oocytes expressing red cell CHIP28 protein. Science.

[42] Deen P.M., Verdijk M.A., Knoers N.V., Wieringa B., Monnens L.A., van Os C.H., van Oost B.A. (1994). Requirement of human renal water channel aquaporin-2 for vasopressin-dependent concentration of urine. Science.

[43] Konno N., Hyodo S., Yamaguchi Y., Matsuda K., Uchiyama M. (2010). Vasotocin/V2-type receptor/aquaporin axis exists in African lungfish kidney but is functional only in terrestrial condition. Endocrinology.

[44] Nishimura H., Fan Z. (2002). Sodium and water transport and urine concentration in avian kidney. Symp. Soc. Exp. Biol..

[45] Suzuki M., Shibata Y., Ogushi Y., Okada R. (2015). Molecular machinery for vasotocin-dependent transepithelial water movement in amphibians: Aquaporins and evolution. Biol. Bull..

[46] Mancera J.M., Martínez-Rodríguez G., Skrzynska A.K., Martos-Sitcha J.A. (2018). Osmoregulatory role of vasotocinergic and isotocinergic systems in the gilthead sea bream (*Sparus aurata* L). Gen. Comp. Endocrinol..

[47] Aoki M., Kaneko T., Katoh F., Hasegawa S., Tsutsui N., Aida K. (2003). Intestinal water absorption through aquaporin 1 expressed in the apical membrane of mucosal epithelial cells in seawater-adapted Japanese eel. J. Exp. Biol..

[48] Martínez A.-S., Cutler C.P., Wilson G.D., Phillips C., Hazon N., Cramb G. (2005). Regulation of expression of two aquaporin homologs in the intestine of the European eel: Effects of seawater acclimation and cortisol treatment. Am. J. Physiol. Regul. Integr. Comp. Physiol..

[49] Kim Y.K., Watanabe S., Kaneko T., Huh M.D., Park S.I. (2010). Expression of aquaporins 3, 8 and 10 in the intestines of freshwater- and seawater-acclimated Japanese eels Anguilla japonica. Fish. Sci..

[50] Deane E.E., Luk J.C., Woo N.Y. (2011). Aquaporin 1a expression in gill, intestine, and kidney of the euryhaline silver sea bream. Front. Physiol..

[51] Giffard-Mena I., Boulo V., Abed C., Cramb G., Charmantier G. (2011). Expression and localization of Aquaporin 1a in the sea-bass (*Dicentrarchus labrax*) during ontogeny. Front. Physiol..

[52] Yang J., Zhang J., Wei K., Shen B. (2021). Cloning and expression of four aquaporin homologs from the Chinese black sleeper (*Bostrychus sinensis*): The effects of salinity acclimation. Biochem. Genet..

[53] Cutler C.P., Cramb G. (2001). Molecular physiology of osmoregulation in eels and other teleosts: The role of transporter isoforms and gene duplication. Comp. Biochem. Physiol. A Mol. Integr. Physiol..

[54] Tipsmark C.K., Sørensen K.J., Madsen S.S. (2010). Aquaporin expression dynamics in osmoregulatory tissues of Atlantic salmon during smoltification and seawater acclimation. J. Exp. Biol..

[55] Choi Y.J., Shin H.S., Kim N.N., Cho S.H., Yamamoto Y., Ueda H., Lee J., Choi C.Y. (2013). Expression of aquaporin-3 and -8 mRNAs in the parr and smolt stages of sockeye salmon, *Oncorhynchus nerka*: Effects of cortisol treatment and seawater acclimation. Comp. Biochem. Physiol. A Mol. Integr. Physiol..

[56] Ma T., Verkman A. (1999). Aquaporin water channels in gastrointestinal physiology. J. Physiol..

[57] Cutler C.P., MacIver B., Cramb G., Zeidel M. (2022). Aquaporin 4 is a ubiquitously expressed isoform in the dogfish (*Squalus acanthias*) shark. Front. Physiol..

[58] Ma Q., Liu X., Li A., Liu S., Zhuang Z. (2020). Effects of osmotic stress on the expression profiling of aquaporin genes in the roughskin sculpin (*Trachidermus fasciatus*). Acta Oceanol. Sin..

[59] Kumari S.S., Varadaraj K. (2009). Intact AQP0 performs cell-to-cell adhesion. Biochem. Biophys. Res. Commun..

[60] Varadaraj K., Kumari S.S. (2018). Lens Aquaporin 0 (AQP0) interacts with negatively charged lipids in the fiber cell membrane to promote cell-to-cell adhesion. Inv. Ophthalmol. Vis. Sci..

[61] Froger A., Clemens D., Kalman K., Németh-Cahalan K.L., Schilling T.F., Hall J.E. (2010). Two distinct Aquaporin 0s required for development and transparency of the zebrafish lens. Investig. Ophthalmol. Vis. Sci..

[62] Chauvigné F., Zapater C., Stavang J.A., Taranger G.L., Cerdà J., Finn R.N. (2015). The pH sensitivity of Aqp0 channels in tetraploid and diploid teleosts. FASEB J..

[63] Chauvigné F., Fjelldal O.G., Cerdà J., Finn R.N. (2016). Auto-adhesion potential of extraocular Aqp0 during teleost development. PLoS ONE.

[64] Engelund M.B., Madsen S.S. (2011). The role of aquaporins in the kidney of euryhaline teleosts. Front. Physiol..

[65] Kim Y.K., Ideuchi H., Watanabe S., Park S.I., Huh M., Kaneko T. (2008). Rectal water absorption in seawater-adapted Japanese eel *Anguilla japonica*. Comp. Biochem. Physiol. A Mol. Integr. Physiol..

[66] Martos-Sitcha J.A., Wunderink Y.S., Gozdowska M., Kulczykowska E., Mancera J.M., Martínez-Rodríguez G. (2013). Vasotocinergic and isotocinergic systems in the gilthead sea bream (*Sparus aurata*): An osmoregulatory story. Comp. Biochem. Physiol. A Mol. Integr. Physiol..

[67] Blom N., Sicheritz-Pontén T., Gupta R., Gammeltoft S., Brunak S. (2004). Prediction of post-translational glycosylation and phosphorylation of proteins from the amino acid sequence. Proteomics.

[68] Gouw M., Michael S., Sámano-Sánchez H., Kumar M., Zeke A., Lang B., Bely B., Chemes L.B., Davey N.E., Deng Z. (2018). The eukaryotic linear motif resource—2018 update. Nucleic Acids Res..

[69] Kamsteeg E.J., Deen P.M. (2001). Detection of aquaporin-2 in the plasma membranes of oocytes: A novel isolation method with improved yield and purity. Biochem. Biophys. Res. Commun..

