# Supplementary material for: Neuropeptide Regulation of Aquaporin Intracellular Trafficking in Marine Teleost Rectal Enterocytes
Source: Int J Mol Sci. 2026 Oct 22;27(21):9427. doi: 10.3390/ijms27219427 (PMC13762709; doi:10.3390/ijms27219427)
Supplement: Supplementary file 1 [file ijms-27-09427-s001.zip › ijms-4609750-supplementary.pdf]

Supplementary Figures

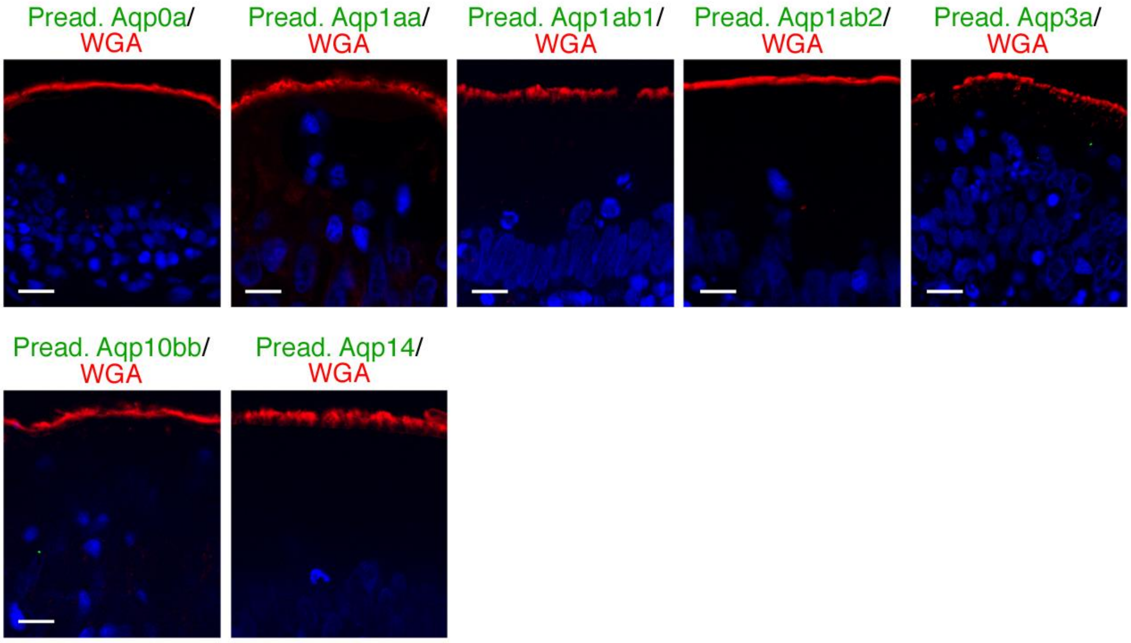

Fig. S1. Control sections for Aqp0a, -1aa, -1ab1, -1ab2, -3a, -10bb and -14 immunostaining. Representative merged images of section from rectal explants incubated with paralog-specific antisera preabsorbed with the respective immunizing peptides and labelled with WGA. Scale bars, 10 µm.

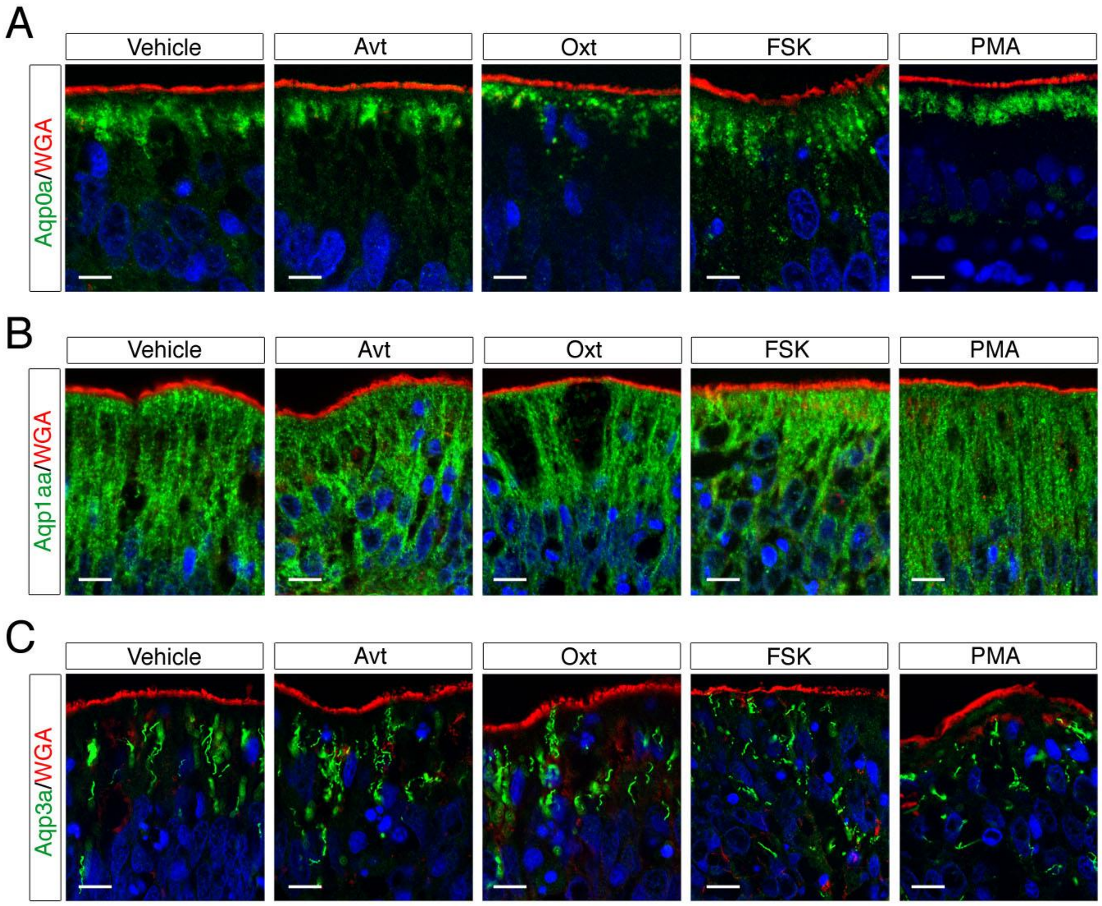

Fig. S2. Trafficking of Aqp0a, -1aa and -3a in rectal enterocytes is not regulated by Avp, Oxt, PKA or PKC. Representative merged images of the immunostaining of Aqp0a (A), - 1aa1 (B) and -3a (C) (green color) and WGA labelling (red) in enterocytes from rectal explants exposed to 10 µM Avp or Oxt, 100 µM FSK, 100 nM PMA, or vehicle (0.5% DMSO) *in vitro*. In all sections, the nuclei were counterstained with DAPI (blue color). Scale bars, 10 µm.
